# Ten Simple Rules for Developing a MOOC

**DOI:** 10.1371/journal.pcbi.1005061

**Published:** 2016-10-20

**Authors:** David T. Manallack, Elizabeth Yuriev

**Affiliations:** Faculty of Pharmacy and Pharmaceutical Sciences, Monash Institute of Pharmaceutical Sciences, Monash University, Parkville, Victoria, Australia; Whitehead Institute, UNITED STATES

If you have not heard of the word MOOC, it refers to **M**assive **O**pen **O**nline **C**ourses, and their appeal has raised the interest of most tertiary institutions. MOOCs have rapidly become “the new black” for online learning [1]. The first course emerged in 2008 from the University of Manitoba (http://cck11.mooc.ca/), and the term MOOC was coined at that time by Dave Cornier (http://davecormier.com/edblog/2008/10/02/the-cck08-mooc-connectivism-course-14-way/). An ever-increasing number of courses from major universities with a range of course providers is now established, and new ones are coming out on a weekly basis.

At Monash University, we recently completed and delivered a MOOC (the “Science of Medicines”) through the FutureLearn platform (https://www.futurelearn.com/courses/the-science-of-medicines). The course has been very successful (total enrollment of 28,500 learners) and has run five times. To facilitate the development of MOOCs, we have put together a set of ten simple rules based on our experiences to give you tips on what to look out for and what to avoid. It must be said, however, that this is not an exercise for the faint-hearted, as MOOC development entails a considerable amount of work. Within the world of MOOCs, there are both introductory and intermediate level courses. Our rules primarily relate to the former and are aimed at academics; however, many of the principles are in common.

## Rule 1: Educator Mission—Establish the Purpose

The question that needs to be asked is, “Why do you want to develop a MOOC?” Is it merely fashionable to have one or is there a serious educational imperative? Certainly a major appeal of MOOCs is that they provide education to a multitude of people across the planet (usually without cost). Instead of lecturing to a class of 100 individuals, you can reach many thousands of people, often on a topic close to your heart. One should also keep in mind the broad diversity of participants who will differ considerably in their educational background, age, and culture. In some cases, the MOOC is oriented to showcasing the educational strengths of an institution and represents, in effect, a branding exercise. Academics called in to create the MOOC need to debate the learning objectives and its purpose for their own clarity and motivation.

## Rule 2: What Is a MOOC?—Experience a MOOC Firsthand

How do they operate? One simple way of exploring these online offerings is to enroll in a MOOC and do some reconnaissance. Given that all MOOCs suffer from a rapid drop-off in numbers throughout the course, you need to consider how to maintain the interest of the participants. Involving yourself in a MOOC will let you understand what works and which platforms are appealing.

## Rule 3: Select a MOOC Provider

Going it alone will probably involve a lot of work and likely won’t play to the strengths of your organization. As such, you should pin yourself to an existing platform/provider (e.g., Coursera, edX, FutureLearn. For the full list, see https://www.mooc-list.com/). Start by exploring, and focus on what the provider offers to the MOOC developers. For example, do they give advice or assistance on structuring a MOOC, quality control, captioning, and hosting services? Once a provider is chosen, a dialogue can be started to gather details and determine whether there is an alignment between their organization and your educational vision. If legal agreements are needed (e.g., royalty sharing), then an appropriate amount of time should be allocated for procuring advice and finalizing contracts.

## Rule 4: Decide on Subject Matter

If the MOOC is showcasing your institution, then this already narrows the subject area. The MOOC can’t teach an entire degree in a matter of a few weeks, so keeping it enjoyable, punchy, and interesting is vital. The decision on the topic needs considerable thought to keep future participants engaged. Again, the educational vision needs to drive this to carefully select the material to deliver. Personal passions to teach the world everything close to your heart will need to be curtailed and put in terms of “what specifically would enlighten them.” The mission, objectives, and specific structure of the MOOC should be carefully defined. This will also help educators who will be invited to design the MOOC to buy into the development process.

## Rule 5: Determine Governance

From the earliest murmurings of your MOOC development, several people will have been involved. For efficiency, there needs to be an established team with clearly defined roles. This will include several academics to both write and present material, a videographer and video editor, a graphics artist, project manager, text editor, and solicitor (where needed). Budgets and schedules must be assembled including tasks to be completed, meeting dates, etc. Even more important is communication, via email and shared document areas. MOOC providers can be extremely useful to map out tasks, ensure quality control, monitor intellectual property, facilitate dialogue, and set up deadlines. The lines of communication also need to be stated, such as who will liaise with the MOOC provider. Having a release date certainly sharpens the mind.

## Rule 6: Design Your MOOC

Clearly the design task itself is a huge topic and cannot be fully covered in this set of rules. Depending on the MOOC provider, they are likely to have a set format for their courses. This is an advantage, as it specifies valuable guidelines on videos, quizzes, discussion boards, polls, and so forth. Each person generating the teaching material then works to a common framework. This not only benefits the educators producing the material but greatly helps learners who get used to a particular style. Estimated time commitment for the learner is another vital consideration for the design to render the MOOC enjoyable and achievable.

One more thing to consider is what will set your MOOC apart from the rest. What is special about your team? For example, in our “Science of Medicines” MOOC, we have used videos of characters inhabiting a fictional village named Pharmville to start a conversation about a range of ailments and corresponding treatments. Learners could relate to these characters and were motivated to participate in discussions sharing personal experiences. We also decided not to solely use a standard multiple-choice question approach to get learners to revise. Instead, we invited them to do crosswords, and this turned out to be an engaging and effective revision technique.

Finally, the design should consider the level at which the course is pitched. MOOC participants will have a wide educational background, and with such a diverse set of people, there is a need to carefully explain concepts, provide additional resource material, and avoid jargon. If not—they will soon move on. The language used and delivery style must be well crafted and edited by a professional to ensure it is consistent and understandable. Captioning is another important resource for non-English speaking participants or those with hearing impairments and will require careful checking to avoid errors. Employing professional videographers with graphics skills also facilitates the entire process. If you are filming multiple sections of the MOOC on one day, it is advisable that you have multiple changes of wardrobe so that keen-eyed participants notice the change of clothes.

## Rule 7: Pilot Test Your MOOC

Quality control is the job of all people involved and will be heavily scrutinized by the provider. Consistency of style, graphics, and cross-examination of material will help, but errors are always made. The aim of pilot testing is to pick up problems before releasing the MOOC to the public. By taking a global view of a MOOC, the pilot test (preferably by lay people) can get an overall idea of the course. Not only does this pick up inconsistencies and errors, it can also gauge the overall merit of the course.

## Rule 8: Promote the MOOC

The MOOC provider will be your partner here to promote the MOOC with an international outlook. Within your own institution, there must also be a strategy to inform people at a national level with press releases and through alumni networks. Given the volume of work that went into developing the MOOC and the passion involved, you need bang for your buck. Promotion also harks back to the purpose of the course. Is it to showcase the institution or is it about education for the masses? Or both? Either way, the word needs to get out there about this fantastic new MOOC.

## Rule 9: Manage the MOOC

The moment you launch your MOOC, participants will start “talking.” The discussion forums will need to be moderated, and other activities will require attention. Communication channels and contact people need to be fully functional at this time. Some elements of the MOOC may require ongoing refinement. But more importantly, questions from learners and debates between them will likely arise. This will require further moderation and regular feedback. Educators, responsive and eager to provide feedback, make for happy and appreciative learners to avoid the MOOC appearing “dead.” And remember, there is always someone on the planet that is wide awake and learning from your MOOC—it is a 24 hr/day enterprise.

Another foible is that the developers, as experts in their field, may have inadvertently introduced difficult concepts that require further clarification. By quickly putting together short videos while the MOOC is running, these misunderstandings can be mitigated (e.g., the YouTube channel associated with our MOOC https://www.youtube.com/channel/UCGp8ENReT_kw6YRPm33MIVQ).

## Rule 10: MOOC Postmortem—Debrief

As the MOOC approaches the release date, the MOOC provider can continually communicate enrollment numbers. They will ultimately inform the institution about various metrics such as demographics, length of time in the course, completion rates, and so forth. These can be used to compare against other offerings and be discussed internally by your MOOC team. Data concerning the demographics of the participants at the start and the end of the course provides an insight into those who persist through the entire MOOC. Of interest is their prior educational experience, which can help in the fine tuning of subject areas that were found difficult to assimilate.

Of prime importance is the need to recognize the involvement of educators, as these exercises take up a considerable amount of time that detracts from other activities. Lastly, the MOOC doesn’t die there. If successful and well received, it is expected that the MOOC will be rerun periodically, which again means moderators need to be summoned.

Incorporating MOOC materials into your regular courses is another benefit following its development. Moreover, the resources can be used to develop graphics material and video vignettes to promote your courses to future undergraduates.

Overall, developing a MOOC is onerous; but in a team environment and with a structured framework, they provide excellent motivation for creating engaging teaching materials designed to enlighten as many as possible.
